# Determinants of renal recovery and mortality inpatients undergoing continuous renal replacement therapy in the ICU

**DOI:** 10.1186/2197-425X-3-S1-A54

**Published:** 2015-10-01

**Authors:** PJ Gleeson, IA Crippa, DJ Sexton, V Fontana, F Taccone, J Creteur, JL Vincent

**Affiliations:** Hôpital Erasme, Service des Soins Intensifs, Brussels, Belgium; Saolta Hospital Group, Nephrology, Galway, Ireland; Beaumont University Hospital, Nephrology, Dublin, Ireland

## Introduction

Continuous Renal Replacement Therapy (CRRT) is commonly used in critically ill patients with acute kidney injury (AKI). However, factors which influence outcome and the optimal way to integrate CRRT into the care of these patients needs to be better defined[1].

## Objectives

We aimed to identify factors associated with mortality and renal recovery among patients requiring CRRT in the ICU. We also measured residual creatinine clearance during CRRT.

## Methods

Electronic medical records of patients requiring CRRT during their stay in our 35-bed medical-surgical ICU (January 2012-December 2013) were reviewed. Patients were excluded if they were dialysis-dependent prior to admission, started on CRRT for toxin removal only, transferred to another hospital while on CRRT or died within 24 hours of admission. Binary logistic regression was performed to identify independent variables significantly associated with mortality and renal recovery after adjusting for age, gender, APACHE II score and chronological time to starting CRRT. Creatinine, net fluid balance and ultrafiltration were adjusted for weight; serum urea was adjusted for gastro-intestinal bleeding and steroid use.

## Results

Among the 157 patients treated with CRRT, 67 (43%) recovered renal function and 80 patients (51%) survived. The median [IQR] APACHE II score on ICU admission was 23 [17-26], the total ICU length of stay was 10 [5-18] days and the duration of CRRT was 6 [3-9] days. Adjusted predictors of ICU mortality were sepsis (OR 2.5; 95% CI 1.3-4.9 - p = 0.008), ARDS (OR 2.1; 1.0-4.4 - p = 0.049) and increasing net fluid balance (OR 1.01; 1.00-1.01 - p= < 0.001); higher creatinine at the time of starting CRRT was independently associated with lower ICU mortality (OR 0.96; 0.04-0.98 - p= < 0.001). Serum urea at the start of CRRT was not associated with mortality. Among patients that did not die on CRRT, factors that predicted failure of renal recovery were a history of chronic kidney disease (OR 0.38, 0.16-0.91 p = 0.03) and increasing ultrafiltration (OR 0.966; 0.94-0.99 - p = 0.015). Furosemide dose had no bearing on renal recovery. Residual creatinine clearance measured during the 24-48 hours prior to stopping CRRT had a greater AUC (0.90, 95%CI 0.84-0.97) for predicting freedom from RRT than urine output (0.87, 0.80-0.95) but was not statistically significant (p = 0.35).

## Conclusions

The mortality rate of patients requiring CRRT was high and only a minority of them were independent of RRT after discharge. Higher serum creatinine level at the time of starting CRRT was associated with survival, suggesting it may be advantageous to start CRRT “late”. Greater positive fluid balance predicted death however in those who survived, increased ultrafiltration was associated with worse renal outcomes, suggesting that maintenance of adequate blood volume is of major importance for the kidneys.
